# Expression of CD44 and Its Correlation With Clinicopathological Factors in Oral and Oropharyngeal Carcinoma: A Retrospective Immunohistochemical Study

**DOI:** 10.7759/cureus.81274

**Published:** 2025-03-27

**Authors:** Mayank Singh, Pallavi Agrawal, Eshan Sharma, Jitendra S Yadav, Chandni Sethi, Neetu Sharma

**Affiliations:** 1 Department of Pathology, Maharani Laxmi Bai Medical College, Jhansi, IND; 2 Department of Otorhinolaryngology, Maharani Laxmi Bai Medical College, Jhansi, IND; 3 Department of Pathology, Sarojini Naidu Medical College, Agra, IND

**Keywords:** cancer stem cells, cd44, immunohistochemistry, oral cancers, oropharyngeal squamous cell carcinoma (scc)

## Abstract

Background: CD44 is a cancer stem cell marker (CSC), which plays a crucial role in cell-to-cell and cell-to-matrix adhesion and is responsible for angiogenesis, invasion, and migration in oral and oropharyngeal cancer.

Objectives: The primary objective of the study was to assess the expression of CD44 in oral and oropharyngeal carcinoma using immunohistochemistry and compare the expression of CD44 in different histological grades of oral and oropharyngeal squamous cell carcinoma (OOSCC). The secondary objective was to correlate the prognosis with the expression.

Material and methods: Based on the histopathological diagnosis, we retrieved OOSCC paraffin-embedded blocks from the archives of the Department of Pathology, Maharani Laxmi Bai Medical College, Jhansi (UP), India, reported from June 2023 to July 2024. We evaluated sections from 31 paraffin blocks of well-differentiated OOSCC, 33 moderately differentiated OOSCC, and six poorly differentiated OSCC for CD44 immunostaining. The immunohistochemical expression of CD44 was studied in these tissues and graded based on the intensity of staining. The difference in immune expression of CD44 between different grades was analyzed. The association of CD44 with all the variables was calculated and interpreted by applying the chi-square test and a p-value <0.05 was taken as significant*. *All statistical analysis was performed and done via computer-based IBM SPSS Statistics for Windows, version 26.0 (released 2019, IBM Corp., Armonk, NY).

Results: We found that all 70 cases of OOSCC showed immunopositivity with CD44 with varying staining intensities. In our study, 33 cases exhibited strong staining intensity, 31 cases were of moderate staining intensity, and six cases exhibited weak to poor staining intensity. Out of 31 well-differentiated of OOSCC, 29 case showed strong staining intensity and two cases showed moderate staining intensity. However, in 33 cases of moderately differentiated SCC, only four cases showed strong staining intensity, and none of the poorly differentiated OOSCC cases showed strong staining intensity. Our findings of grade versus staining intensity of CD44 expression by tumor cells in OOSCC came out to be statistically significant (p < 0.05).

Conclusion: The expression of CD44 was noted to decrease from well-differentiated to poorly differentiated OSCC.

## Introduction

Neoplasia or, in simple terms, cancer is often described as a disorder of the cell cycle. While this perspective suggests that tumors typically involve defects in cell cycle regulation, it does not imply that oncogenesis occurs only due to defects in cell cycle or because of certain oncogenes.

The progression to malignancy also appears to necessitate abnormalities in processes, such as cell death mechanisms, and interactions between cells and their environment (cell-cell and cell-matrix), which collaborate with cell cycle dysfunctions.

Head and neck cancer, commonly prevalent in Asia and notably in India with over 200,000 new cases annually, contributes significantly to global cancer statistics. Approximately 7.8% of the global burden is contributed by India [1]. Oral cancer alone constitutes 30% of all cancers in India [2], primarily as squamous cell carcinoma (SCC) affecting the oral cavity. A substantial proportion (>60%) of patients present with advanced disease stage [3]. Globally, head and neck cancers rank sixth overall, eighth among men, and fifth among women in frequency [4].

Oropharyngeal SCC, also known as throat cancer, are malignancy affecting the pharynx's middle part. Above 90% of these carcinomas are SCCs, predominantly lining the oropharynx [5].

Risk factors for oral and oropharyngeal cancers include oral HPV infection (linked to practices like oral sex and open-mouthed kissing) [6,7,8], whereas less common risk factors include tobacco smoking, alcohol consumption, poor diet, betel quid chewing, exposure to factors like asbestos, and mutations in genes such as P53 and CDKN2A (p16) [9].

Despite advancements in cancer therapies, recurrence can occur due to residual cancer stem cells (CSCs) left behind after treatment. CSCs are specialized groups of cells within tumors capable of self-renewal and tumorigenesis and are also known as tumor-initiating cells (TICs). Initially identified in AML in 1994 by workers John Dick and his colleagues [10], these cells have since been recognized in various human cancers such as CNS tumors, multiple myeloma, colorectal cancer, prostatic carcinoma, head and neck carcinoma, melanocytic tumors, hepatocellular malignancy, hepato-pancreatic cancer, and pulmonary carcinoma.

Originating from normal stem cells, CSCs acquire various mutations distinguishing them from their non-cancerous counterparts.

The key distinction between "stem cells" versus "cancer stem cells" lies in the latter's heightened tumorigenic potential and self-renewal abilities as TICs.

CD44 is a prominent surface marker on CSCs [11,12] and a promising therapeutic target across various cancers due to its role in cell-to-cell interactions, cell-to-matrix interactions, and activation of different signaling pathways. [13]

It is widely expressed on normal hematolymphoid cells, fibroblasts, myofibroblasts, and numerous cancer cells, especially in SCCs of various organs like the oral cavity, cervix, and vulva. CD44 is also expressed in melanomas, breast carcinomas, and prostate cancer [14].

CD44, an integral glycoprotein of the cell membrane [15], plays an essential role in cell migration and adhesion. It is recognized as a significant marker for identifying CSCs in OOSCC [16]. CD44 interacts with several ligands, with hyaluronic acid (HA) being the most prominent [17]. HA, composed of repeating disaccharide units, is a key component of the extracellular matrix and is thought to facilitate enhanced cell motility by creating a matrix with low resistance [18,19]. CD44 exists in numerous isoforms, with the standard CD44 (CD44st) being the most prevalent. Notably, CD44 is considered an early indicator of malignancy, and its isoform expression varies between normal and tumor cells, highlighting its roles in both physiological and pathological contexts [20].

The overexpression of CD44 has been linked to poorer outcomes in SCCs of the oral cavity. Given that oral and oropharyngeal cancers contribute substantially to global cancer mortality, early detection and timely treatment are critical for improving survival rates and reducing mortality. Numerous biomarkers have been proposed as prognostic indicators for OSCC.

This present study aims to investigate the expression of CD44 in oral and oropharyngeal carcinoma and its correlation with clinicopathological factors.

## Materials and methods

General study details

This retrospective observational study was conducted at the Department of Pathology at Maharani Laxmi Bai (MLB) Medical College, Jhansi, India, from June 2023 to July 2024. Ethical clearance was obtained from the institutional review board and ethical committee (2209/IEC/I/2023-2024) on May 31, 2023. For the retrospective analysis of tissue-embedded blocks, the institutional ethics committee waived the informed consent. 

Participants

We collected well-differentiated OOSCC, moderately differentiated OOSCC, and poorly differentiated OOSCC cases based on the inclusion and exclusion criteria. We included blocks with sufficient tissue and proper demographic and clinical data of primary untreated cases of OOSCC in the study. Recurrent cases, regional lymph node metastasis, distant metastasis, and therapeutic interventions were excluded from the study. Normal oral epithelium was used as a positive control in our study.

Study methodology

Formalin-fixed paraffin-embedded tissue blocks were analyzed from June 2023 to July 2024 from the records of the Department of Oral Pathology. A total of 70 blocks of histopathologically diagnosed cases of OOSCC were retrieved based on the convenience sampling method. Based on the World Health Organization (WHO) classification of tumors, 31 well-differentiated OOSCC, 33 moderately differentiated OOSCC, and six poorly differentiated OOSCC were selected for the immunohistochemical staining. Demographic and clinical details were also retrieved for these 70 cases.

Immunohistochemistry

Tumor blocks were selected after a review of corresponding H&E-stained slides to select and confirm representative areas. Immunohistochemistry procedures were performed in a Ventana benchmark automatic slide staining system according to the manufacturer’s instructions with minor modifications.

First, four-micron sections on poly-L-lysine-coated slides were cut, followed by deparaffinization with an EZ prep solution at 75 degrees for 30 minutes. Antigen retrieval was performed using CC1 (cell conditioner solution) at 95-100 degrees for 60 minutes. Endogenous peroxidase blocking was done by incubation with an ultraView inhibitor (3% H_2_O_2_) for four minutes.

Primary antibody (Bioheaven 360 RTU anti-CD44 rabbit monoclonal primary antibody) was applied and incubated for 32 minutes at 37 °C temperature followed by the application of secondary antibody and incubating for eight minutes at 37 °C temperature. Then, eight drops of DAB chromogen and one drop of DAB H_2_O_2_ were applied and incubated for eight minutes. Moreover, DAB enhancer (copper sulfate) was added and incubated for four minutes. Counterstaining was done with hematoxylin, followed by examination under a light microscope from 4x to 40x magnification.

Evaluation of staining intensity

The most representative areas of tumors were selected for scoring the immune staining pattern and intensity of CD44. The staining pattern was membranous in the budding tumor nests of OOSCC and in the basal and suprabasal cells of normal mucosa. The intensity of the CD44 staining was graded as weak (low staining intensity), moderate (intermediate staining intensity), and strong (high staining intensity) in all 70 tissue sections.

Scoring was done as tumor proportion score, which represents the proportion (%) of CD44-positive tumor cells relative to the total number of viable tumor cells x100 according to the criteria of Simionescu C et al. [20].

Statistics

As this was a retrospective study, we did not calculate the sample size a priori. The samples were selected using the convenience sampling method. Data obtained were recorded into a Microsoft Excel spreadsheet (Microsoft Corporation, USA). Qualitative data were expressed as frequency (f) along with percentages (%). The association of CD44 with all the variables was calculated and interpreted by applying the chi-square test, and a p-value <0.05 was taken as significant. All statistical analysis will be performed and done via the computer-based IBM SPSS Statistics for Windows, version 26.0 (released 2019, IBM Corp., Armonk, NY). Charts were prepared using Microsoft Excel 2007.

## Results

Based on the inclusion and exclusion criteria, 70 cases of OOSCC were considered from June 2023 to July 2024. Histological examination was performed, and cases were graded as well-differentiated, moderately differentiated, or poorly differentiated SCC according to Broder’s (1920) grading criteria, which are mainly based on the degree of differentiation and amount of keratinization. According to grading, cases were categorized into three groups.

Group 1 (n = 31) comprised well-differentiated OOSCC patients, group 2 (n = 33) comprised moderately differentiated OOSCC patients, and group 3 (n = 6) comprising poorly differentiated OOSCC patients, as shown in Table 1.

**Table 1:** Distribution of cases according to histological grading

Group	Grade	No. of cases	Histological grading
Group 1	Grade 1	31	Well-differentiated oral and oropharyngeal squamous cell carcinoma
Group 2	Grade 2	33	Moderately-differentiated oral and oropharyngeal squamous cell carcinoma
Group 3	Grade 4	6	Poorly-differentiated oral and oropharyngeal squamous cell carcinoma

Among the total cases, the majority of cases belonged to 41-60 years of age (33/70). Most cases were males (58/70) and belonged to urban areas (46/70). Most of the cases were of farmers (40/70) who had exposure to pesticides and harmful carcinogenic chemicals, while the rest of the cases (30/70) were of other occupations. In our study, most cases had one or more combined habits of either tobacco chewing, smoking, or alcohol.

In group 1, 11/31 cases were the habit of tobacco and smoking, 7/31 were only tobacco chewing, and 6/31 were tobacco with alcohol habits. Only 3/31 cases were habits of alcohol and smoking alone. Out of 33 cases from group 2, 13 cases were habits of tobacco and smoking, seven cases were habit of only smoking, and four cases were habits of tobacco and alcohol. Out of six cases from group 3, two cases were habits of tobacco and smoking, while only one case from each tobacco chewing, smoking, and tobacco with alcohol habit.

Most cases (38/70) showed that buccal mucosa was the most common site of the lesion, followed by the hard palate (11/70). The remaining cases had primary lesions on other sites of the oral cavity, which included the tongue, epiglottis, pyriform fossa, vocal cords, alveolar ridge, pharyngeal wall, and aryepiglottic folds.

Out of 31 cases from group 1, the site of lesion in 18 cases was found in the buccal mucosa, five cases in the hard palate, three cases at the pharyngeal wall, and two at the retromolar trigone.

Out of 33 cases from group 2, the site of lesion was buccal mucosa in 16 cases, in five cases hard palate, three cases alveolar ridge, and two cases each at epiglottis and retromolar trigone. Out of six cases from group 3, the site of lesion was buccal mucosa in four cases and one each in the hard palate and pyriform fossa. The demographic and clinical details of different study groups are detailed in Table 2.

**Table 2:** Demographic and clinical details of patients with different grades of oral and oropharyngeal squamous cell carcinoma (OOSCC).

Parameter	Group 1 (n = 31)	Group 2 (n = 33)	Group 3 (n = 6)	Total
Age (years)				
20-40	08	12	02	22
41-60	12	17	04	33
>60	11	04	0	15
Sex				
Male	25	29	04	58
Female	06	04	02	12
Locality				
Urban	12	17	04	46
Rural	06	16	02	24
Occupation				
Farmers	18	18	04	40
Others	13	15	02	30
Habits				
Tobacco chewing	07	03	01	11
Tobacco + smoking	11	13	02	26
Alcohol	03	03	00	06
Smoking	03	07	01	11
Tobacco+ alcohol	06	04	01	11
None	1	03	01	05
Site of lesion				
Buccal mucosa	18	16	04	38
Hard palate	05	05	01	11
Other sites	08	12	01	
Total	31	33	06	70

**Figure 1:**
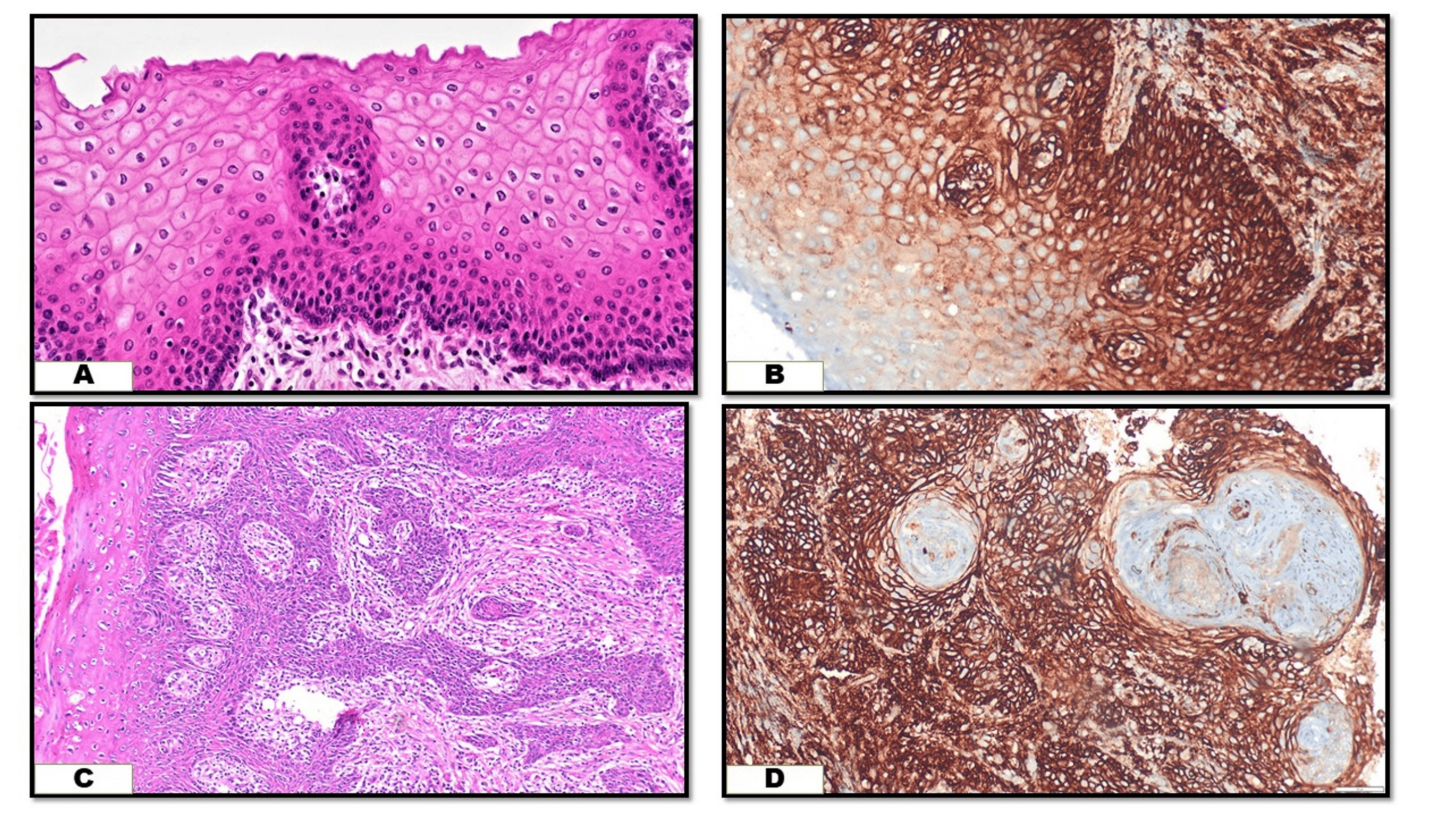
(A) H&E-stained section of normal stratified squamous epithelium of the oral cavity (10x),(B) CD44 expression in the normal epithelium, strong staining intensity in the basal and parabasal layers (10x), (C) H&E-stained section of well-differentiated OOSCC (10x), (D) strong CD44 expression in well-differentiated OOSCC (10x).

**Figure 2:**
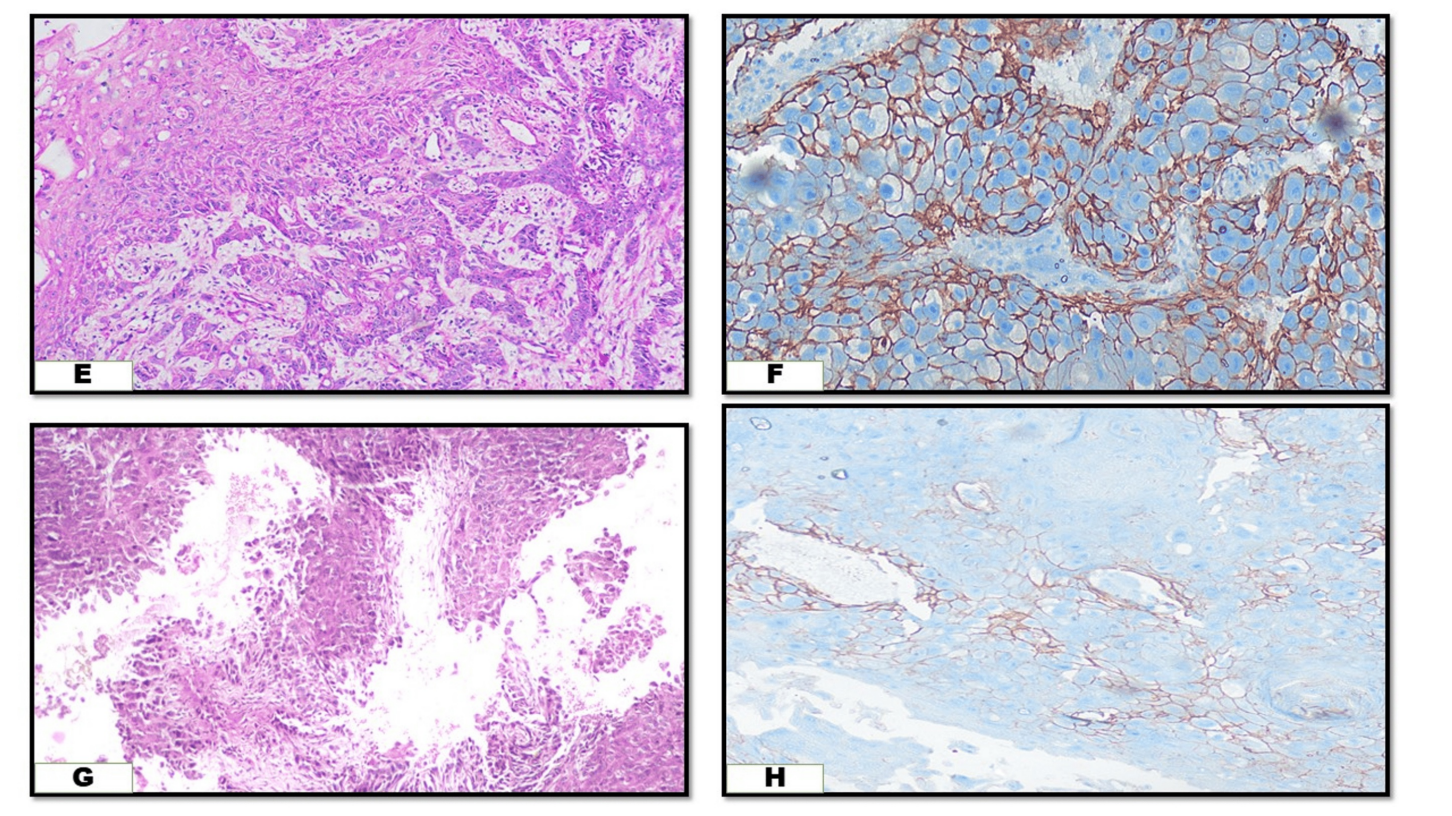
(E) H&E-stained section of MD OOSCC (10x), (F) CD44 expression in MD OOSCC - moderate staining intensity (10x), (G) H&E-stained section of PD OOSCC (40x), (H) CD44 expression in PD OOSCC - weak to absent staining intensity (40x). MD: moderately differentiated, OOSCC: oral and oropharyngeal squamous cell carcinoma, PD: poorly differentiated

CD44 expression was predominantly observed on the membrane in oral cancer tissues. In normal mucosa, positive staining appeared in the basal and parabasal layers, indicating a possible stem cell-like quality in these cells. In well-differentiated OOSCC, CD44 positivity was present in budding tumor cells at the invasive front. Tumor cells surrounding keratin pearls (small, round, keratinized structures characteristic of well-differentiated OOSCC) also showed positive staining. The staining intensity decreased progressively from well-differentiated to poorly differentiated OSCC, as illustrated in Figures 1-2. None of the poorly differentiated OSCC samples exhibited strong CD44 staining. Table 3 outlines the varying staining intensities across different OOSCC grades.

**Table 3:** Distribution of cases according to CD44 staining intensity and association between staining intensity versus grades of oral and oropharyngeal squamous cell carcinoma * chi-squared Test, # - significant < 0.05. WD: well-differentiated, MD: moderately differentiated, PD: poorly differentiated, OOSCC: oral and oropharyngeal squamous cell carcinoma

CD44 staining intensity	Total number of cases	Group 1 (WD OOSCC)	Group 2 (MD OOSCC)	Group 3 (PD OOSCC)	P-value*
Strong	33	29	04	0	<0.001^#^
Moderate	31	02	28	01	
Weak/poor	06	0	01	05	

Out of 31 cases of group 1, 29 had strong staining intensity and the remaining two cases were of moderate staining intensity. In group 2, which consists of 33 cases, 28 cases showed moderate intensity, while four cases had strong intensity with CD44 expression and one case had poor intensity. In group 3, out of six cases, five cases had poor staining intensity and one case was found to be moderate intensity.

On evaluating the percentage of positive tumor cells expressing CD44, we found that in group 1, 13 cases showed 51-75% of positive cells, while in group 2, 14 cases showed 26-50% of positive cells, and in group 3 cases, three cases had 51-75% positive cells and another three cases had >75% positive cells, respectively, as shown in Table 4. 

**Table 4:** Distribution of cases according to percentage of positive tumor cells expressing CD44

Percentage of positive cells	Total	Group 1	Group 2	Group 3
0 (<10%)	0	0	0	0
1 (11-25%)	4	2	2	0
2 (26-50%)	19	5	14	0
3 (51%-75%)	29	13	13	3
4 (>75%)	18	11	4	3
Total	70	31	33	6

## Discussion

Cancer of the oral cavity is the 11th most common cancer globally, with India accounting for approximately 1/5th of all cases and approximately 1/4th of all deaths resulting from oral cancer. These high case fatality rates in India are due to the widespread use of chewing tobacco, smoking, reverse smoking, chewing of betel quid, and areca nut. In addition, some idiopathic cases can be associated with individual genetic factors and environmental influences. Mortality rates in oral cancer are significantly impacted by incidence rates, access to appropriate treatments, site involved, recurrence rates, and potential to metastasize [21]. It has been found that cancer stem cells can initiate and sustain the growth of the tumor cells and have characteristics similar to stem cells, such as self-renewal and multilineage differentiation.

CD44, a versatile cell surface molecule, is crucial in various cellular processes including cellular proliferation, differentiation, migration, angiogenesis, cytokine presentation, chemokine and growth factor receptor interaction, protease docking at the cell membrane, and cell survival. These functions are vital for normal physiology but also play a crucial role in cancer cell pathology.

Our study aimed to examine the expression of CD44 in oral and oropharyngeal carcinoma using immunohistochemistry and compare it across different histological grades. We took 70 cases of OOSCC and graded them into well, moderate, and poorly differentiated carcinomas. We found that the majority of cases across all groups were between 41 and 60 years of age. These findings align with Monteiro et al.'s study [22], which found that a greater number of patients with OSF (73.3%) were below 40 years of age, whereas 63.3% of OSCC cases were above 40 years of age. Similar results were observed in studies by Monterio et al. [23] Margaritescu et al. [24], and Linquist et al. [25], which also showed male predominance. Moreover, similar findings were seen in a study done by Kazmi et al. [26], where the average mean age group of cases with OOSCC was 37.7 ± 9.7 years.

In our study, 46 cases (65.71%) belonged to urban areas while the remaining 24 cases (34.29%) belonged to rural areas. When we subdivided the cases based on occupation and exposure, the majority of the cases were farmers (57.14%) while the remaining cases were mostly outdoor workers. Thus, our study found out that farmers have a much higher incidence of OOSCC probably due to several occupational and environmental factors, including pesticide exposure, sun exposure, tobacco smoking, alcohol use, dietary habits, and living in areas with limited access to healthcare services, leading to delays in the diagnosis and treatment of oral cancers. We also observed that most of the cases belonged to low socioeconomic status (49 cases). This may be related lack of awareness about oral cancer, poor oral hygiene, and financial limitations.

While observing the tobacco and smoking habits, most of the cases of OOSCC were a result of one or more combined habits of either tobacco chewing, smoking, or alcohol consumption. We discovered that 26 cases (37.14%) had a habit of combined tobacco chewing and smoking, whereas 11 cases (15.71%) each had a habit of tobacco chewing alone, smoking alone, and alcohol intake alone. This indicates that the combination of tobacco in any form and alcohol drinking is a major cause of oral cancer and when combined leads to increased epithelial permeability, thereby enhancing exposure to carcinogens from all sorts of tobacco products. These results align with studies and observations noted by Kazmi et al. [26]. Our study found that the most common site involved in OOSCC was the buccal mucosa, accounting for 38 (54.29%) cases, followed by 11 (15.71%) cases with lesions at the hard palate. The remaining cases had lesions in various regions of the oral cavity and oropharynx. Almost similar results were observed in the study by Kazmi et al. [26], where 61.9% of cases had lesions in the buccal mucosa.

Regarding immune expression with CD44, all 70 cases showed immunopositivity with varying staining intensities. Overall, 33 cases exhibited strong staining intensity, 31 cases were of moderate staining intensity, and six cases exhibited weak or poor staining intensity. Our study found that Out of 31 well-differentiated OOSCC, 29 cases showed strong staining intensity, and two cases showed moderate staining intensity. However, in 33 cases of moderately differentiated SCC, only four cases showed strong staining intensity and none of the poorly differentiated OOSCC cases showed strong staining intensity. Our findings align with studies by Nand Kumar H et al. [27], Chakraborty et al. [28], and Kaza et al [29], which also showed a stronger staining intensity in well-differentiated OOSCC and weak to absent staining intensity in poorly differentiated cases.

Our findings of grade versus staining intensity of CD44 expression by tumor cells in OOSCC came out to be statistically significant (p < 0.05) and suggested that the decrease in immune expression with increasing tumor grade is due to the loss of adhesion between cells, which ultimately leads to the easier detachment between cells from their typical rigid and firm configuration and architecture. The different patterns and staining intensities of CD44 expression in different grades of OOSCC were due to the presence of pleomorphic neoplastic cells. Poorly differentiated OOSCC exhibited highly pleomorphic cells, which were prone to easy detachment from their rigid configuration, and thus a weak CD44 immunostaining intensity was recorded.

While evaluating the percentage of positive tumor cells in 70 cases of OOSCC, the majority of cases (29) showed 51-75% of positive tumor cells (score 3), followed by 19 cases with a score of 2 (26-50% of positive tumor cells). Moreover, a higher number of percentage of positive tumor cells were seen in well-differentiated OOSCC. When we studied the n association between the percentage of positive tumor cells and various sociodemographic factors, no significant finding was noted (p > 0.05).

Moreover, no statistical significance was found between the percentage of positive tumor cells and the site of the lesion. Thus, in our study, we found that staining intensity of CD44 in different grades of OOSCC could be an independent prognostic factor. A limitation of the study is that the patients with regional or distant metastasis were not included. Second, the staining pattern was not compared with different grades of dysplasia, and other cancer stem cell markers such as CD133 were also not studied.

## Conclusions

The present study showed the weak immunostaining of CD44 in poorly differentiated OOSCC. It explains the fact the expression is associated with the progression of carcinogenesis and increased chances of metastasis to distant organs. Therefore, further studies targeting cancer stem cells might demonstrate an effective way to accelerate tumor cell death and reduce resistance to chemotherapy.

## References

[1] Dhananjaya S, Aparna K (2014). Current status of cancer burden: global and Indian scenario. Biomed Res J.

[2] (2006). Trends of head and neck cancers in urban and rural India. Asian Pac J Cancer Prev.

[3] Östman J, Anneroth G, Gustafsson H, Tavelin B (1995). Malignant oral tumours in Sweden 1960-1989—an epidemiological study. Eur J Cancer B Oral Oncol.

[4] Lippman SM, Hong WK (2001). Molecular markers of the risk of oral cancer. N Engl J Med.

[5] Lippman SM, Sudbø J, Hong WK (2005). Oral cancer prevention and the evolution of molecular-targeted drug development. J Clin Oncol.

[6] Hamada GS, Bos AJ, Kasuga H, Hirayama T (1991). Comparative epidemiology of oral cancer in Brazil and India. Tokai J Exp Clin Med.

[7] Sankaranarayanan R (1990). Oral cancer in India: an epidemiologic and clinical review. Oral Surg Oral Med Oral Pathol.

[8] Laprise C, Shahul HP, Madathil SA (2016). Periodontal diseases and risk of oral cancer in Southern India: results from the HeNCe Life study. Int J Cancer.

[9] Singh M, Prasad CP, Singh TD, Kumar L (2018). Cancer research in India: challenges & opportunities. Indian J Med Res.

[10] Bonnet D, Dick JE (1997). Human acute myeloid leukemia is organized as a hierarchy that originates from a primitive hematopoietic cell. Nat Med.

[11] Yan Y, Zuo X, Wei D (2015). Concise review: emerging role of CD44 in cancer stem cells: a promising biomarker and therapeutic target. Stem Cells Transl Med.

[12] Dalchau R, Kirkley J, Fabre JW (1980). Monoclonal antibody to a human leukocyte-specific membrane glycoprotein probably homologous to the leukocyte-common (L-C) antigen of the rat. Eur J Immunol.

[13] Iczkowski K (2010). Cell adhesion molecule CD44: its functional roles in prostate cancer. Am J Transl Res.

[14] Misra S, Hascall VC, Markwald RR, Ghatak S (2015). Interactions between hyaluronan and its receptors (CD44, RHAMM) regulate the activities of inflammation and cancer. Front Immunol.

[15] Picker LJ, Nakache M, Butcher EC (1989). Monoclonal antibodies to human lymphocyte homing receptors define a novel class of adhesion molecules on diverse cell types. J Cell Biol.

[16] Isacke CM, Yarwood H (2002). The hyaluronan receptor, CD44. Int J Biochem Cell Biol.

[17] Huet S, Groux H, Caillou B, Valentin H, Prieur A, Bernard A (1950). CD44 contributes to T cell activation. J Immun.

[18] Naor D, Wallach-Dayan SB, Zahalka MA, Sionov RV (2009). Involvement of CD44, a molecule with a thousand faces, in cancer dissemination. Hyaluronan in Cancer Biology.

[19] Franzmann EJ, Reategui EP, Pedroso F (2007). Soluble CD44 is a potential marker for the early detection of head and neck cancer. Cancer Epidemiol Biomarkers Prev.

[20] Wang SJ, Bourguignon LY (2011). Role of hyaluronan-mediated CD44 signaling in head and neck squamous cell carcinoma progression and chemoresistance. Am J Pathol.

[21] Sankaranarayanan R, Ramadas K, Amarasinghe H, Subramanian S, Johnson N (2015). Oral cancer: prevention, early detection, and treatment. Cancer: Disease Control Priorities, Third Edition (Volume 3).

[22] Monteiro R, Hallikeri K, Sudhakaran A (202112). PTEN and α-SMA expression and diagnostic role in oral submucous fibrosis and oral squamous cell carcinoma with concomitant oral submucous fibrosis. J Oral Maxillofac Res.

[23] Monteiro LS, Delgado ML, Ricardo S (2016). Prognostic significance of CD44v6, p63, podoplanin and MMP-9 in oral squamous cell carcinomas. Oral Dis.

[24] Mărgăritescu C, Pirici D, Simionescu C, Stepan A (2011). The utility of CD44, CD117 and CD133 in identification of cancer stem cells (CSC) in oral squamous cell carcinomas (OSCC). Rom J Morphol Embryol.

[25] Lindquist D, Ährlund-Richter A, Tarjan M, Tot T, Dalianis T (20121). Intense CD44 expression is a negative prognostic factor in tonsillar and base of tongue cancer. Anticancer Res.

[26] Kazmi A, Abbas Z, Saleem Z, Haider S, Farooqui WA, Ahmed S (2022). Relation of salivary MMP-8 with oral submucous fibrosis and oral squamous cell carcinoma: a cross sectional analytical study. BMJ Open.

[27] Kumar HN, Vasanthi V, Gunasekaran N, Divya B, Annasamy RK, Krishnan R (2024). Correlation of prognosis of oral squamous cell carcinoma with CD44 expression: a retrospective immunohistochemical analysis. Cancer Res Stat Treat.

[28] Chakraborty S, Suresh TN, Azeem Mohiyuddin SM (2024). Expression of stem cell biomarker CD44 in oral squamous cell carcinoma and its association with lymph node metastasis and TNM staging. J Cancer Res Ther.

[29] Kaza S, Kantheti L, Poosarla C, Gontu S, Kattappagari K, Baddam V (2018). A study on the expression of CD44 adhesion molecule in oral squamous cell carcinoma and its correlation with tumor histological grading. J Orofac Sci.

